# Hydrocele in recurrent acute pancreatitis caused by testicular venous obstruction: A case report of a rare complication (CARE-compliant)

**DOI:** 10.1097/MD.0000000000019738

**Published:** 2020-05-01

**Authors:** Yin-Hsi Chang, Shih-Yen Weng, Sheng-Jie Shiue, Chao-Ling Cheng, Ming-Shun Wu

**Affiliations:** a Medical Education Department, Chang Gung Memorial Hospital, Linkou Medical Center, Taoyuan; b School of Medicine, College of Medicine, Taipei Medical University; c Division of Gastroenterology, Department of Internal Medicine, Wan Fang Hospital, Taipei Medical University; d Research Center for Healthcare Industry Innovation, National Taipei University of Nursing and Health Sciences; e Division of Gastroenterology and Hepatology, Department of Internal Medicine, School of Medicine, College of Medicine; f Integrative Therapy Center for Gastroenterologic Cancers, Wan Fang Hospital, Taipei Medical University, Taipei, Taiwan.

**Keywords:** acute pancreatitis, peripancreatic fluid, scrotal hydrocele, testicular venous obstruction

## Abstract

**Rationale:**

Scrotal swelling is a rare complication of acute pancreatitis. It had been explained by fluid accumulation in scrotum originated from abdomen. Here we demonstrated a case of recurrent pancreatitis with hydrocele caused by impaired testicular venous drainage.

**Patient concerns:**

A 53-year-old man presented with sudden onset epigastric pain after an alcohol binge. Recurrent acute pancreatitis was confirmed by medical history, physical examination, elevated lipase level and abdominal computed tomography (CT) scan. Right scrotal swelling was noticed on the next day.

**Diagnosis:**

The scrotal ultrasonography demonstrated fluid accumulation around the testis and varicocele consistent with scrotal hydrocele. CT scans of the abdomen and pelvis showed encasement of the right testicular vein by pancreatic phlegmon.

**Interventions:**

The patient was subject to Nulla per os, hydration, and opioid analgesics for pancreatitis. No intervention was performed for scrotal swelling.

**Outcomes:**

Hydrocele gradually resolved along with acute pancreatitis.

**Lessons:**

Pancreatic phlegmon compromised testicular venous return which led to scrotal hydrocele and posed a threat to fertility. The study has provided a novel pathologic linkage. This complication should be taken into account.

## Abstract 2

Supplemental Digital Content is available in the text

## 1 Introduction

Acute pancreatitis is associated with several local or systemic complications, which help classify the severity of acute pancreatitis. Well-established complications range from local peripancreatic fluid collections, necrotic collections, pseudocyst formation, wall-off necrosis to systemic organ failure.^[1]^ Local fluid collections can also lead to ascites, pleural effusion, and acute respiratory distress syndrome.^[2,3]^ However, it is extremely rare for patients with acute pancreatitis developing scrotal hydrocele.^[4]^ Herein, we report a case of hydrocele caused by recurrent acute pancreatitis that developed scrotal swelling with rare etiology of right testicular vein compression.

## 2 Case presentation

A 53-year-old man, with 5 episodes of acute alcoholic pancreatitis in recent 2 years, presented to the emergency department with intense epigastric pain radiating to back for 2 days. The pain was sharp in character, which he rated at 10 on a scale of 0 to 10 (with 10 indicating the most severe pain) and the patient had to sit in the knee-chest position in an effort to relieve the pain. He reported alcohol binge the night before onset of pain. No associated fever, vomiting, urinary or bowel symptoms, trauma or procedure history was noted. Initial workup revealed serum lipase level of 1375 IU/L corresponding with the diagnosis of acute pancreatitis. On the 2nd day after admission, the patient developed right scrotal swelling and pain without erythematous change. He recalled similar episodes of swollen right scrotum whenever pancreatitis occurred. Nevertheless, it resolved spontaneously in 1 week once acute pancreatitis subsided.

**Figure 1:**
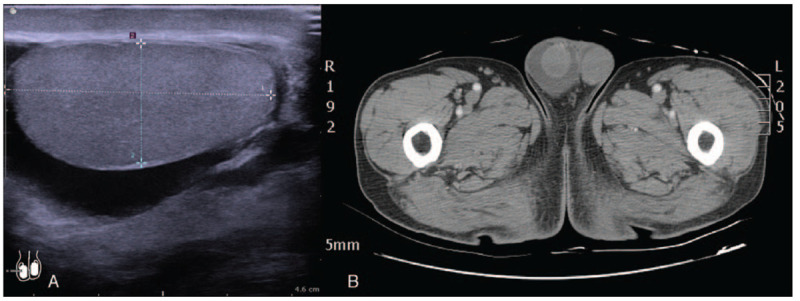
Scrotal imaging. (A) Ultrasonography showed a 41.3 × 18.7 mm egg-shaped testis with anechoic fluid accumulation in the right scrotum. (B) Computed tomography scan revealed enlarged right scrotum with homogeneous fluid collections around the testis without inflammatory, ischemic or necrotic change.

**Figure 2:**
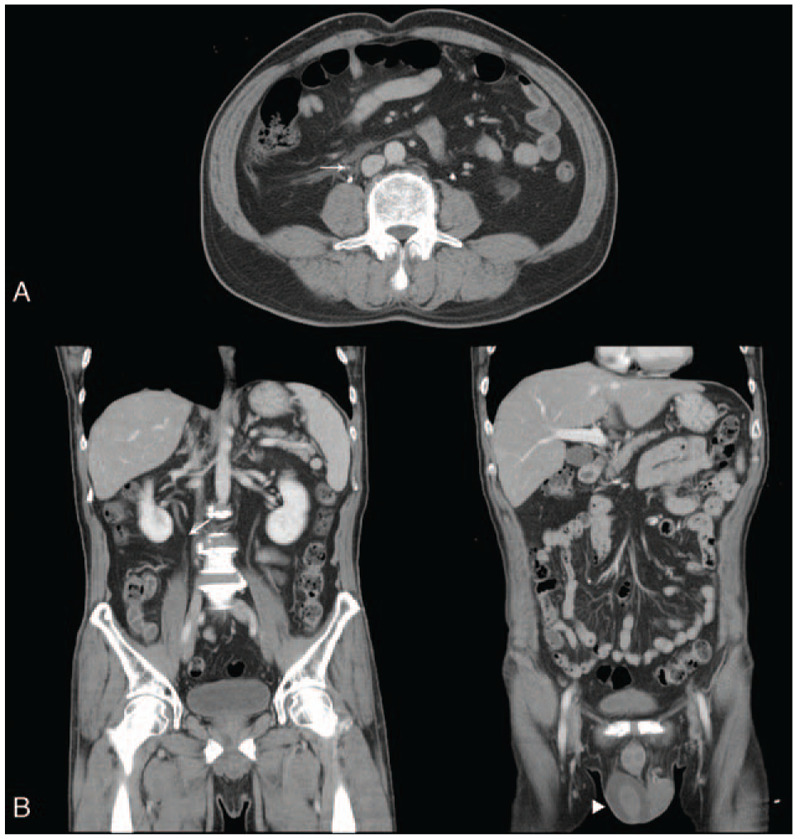
Abdominal computed tomography scan. (A) An axial view showed retroperitoneal fat stranding and minimal fluid at the level below pancreas. Note right testicular vein (arrow) was closely surrounded by adjacent inflammatory tissue. (B) Coronal section demonstrated that the proximal part of right testicular vein (arrow) was obscured by retroperitoneal fluid and edematous tissue. It further led to scrotal hydrocele (arrowhead). There was no massive fluid extending from abdomen to the pelvis. Bilateral psoas muscles were visible.

On examination, there was no tender testis, epididymis, or palpable mass at inguinal region. An ultrasonography of right scrotum revealed marked, anechoic fluid accumulation around the normal testis and periorchium (Fig. 1A). Varicoceles were also noted. An abdominal and pelvic computed tomography (CT) scan demonstrated noncommunicating hydrocele on the right side (Fig. 1B). Instead of massive retroperitoneal fluid extending down through the pelvis, only a small amount of collections and fat stranding occluding the right testicular vein were identified (Fig. 2A, B). The patient received Nulla per os, hydration, and opioid analgesics over the following days. Four days later, the hydrocele gradually diminished. One month after discharge, the following CT confirmed that right testicular vein was free of compression (Supplemental Figure).

## 3 Discussion

In this report, we demonstrated a rare case of recurrent acute pancreatitis with scrotal hydrocele caused by impaired testicular venous drainage instead of fluid tracking from abdomen. Pancreatic hydrocele is a rare complication of acute pancreatitis with 34 cases reported in the literature.^[5–14]^ Previous studies proposed that hydrocele is resulted from peripancreatic fluid in the retroperitoneum tracking through pelvic space, inguinal canal into the scrotum along processus vaginalis.^[5–14]^ Those patients had failure of closure of the processus vaginalis. Consequently, the fluid dissected between the visceral and parietal layers of the tunica vaginalis led to communicating hydrocele. We noted patients with pancreatic hydrocele were mostly alcohol-related among different etiologies of pancreatitis.^[4,7,10,11,15–25]^ Alcohol may increase the production of digestive and lysosomal enzymes, and result in more fluid collections.

In the present case, however, retroperitoneal fluid was scanty, which was different from common causes of pancreatic hydrocele. No strong evidence revealed a direct link between scrotal effusion and retroperitoneal fluid. Instead, blockage of right testicular vein was noted. Figure 1 shows fluid accumulation around the testis that was consistent with hydrocele and Figure 2 demonstrates occlusion of right testicular vein by local phlegmon of pancreas. Aswani and Hira reported a male presented with left varicocele caused by pancreatic pseudocyst compressing the testicular vein.^[26]^ Varicocele on scrotal ultrasonography indicated impaired blood flow by venous obstruction.^[27–29]^ Elevated intravenous pressure can increase the testicular vascular permeability and cause fluid formation.^[30]^ We suggest the effusion of hydrocele in our case mainly resulted from venous congestion due to high pressure. To our knowledge, this is the first case with pancreatic hydrocele that could be attributed to compromised testicular venous return by the pancreatic phlegmon.

Clinically, it is important to differentiate hydrocele from other testicular emergencies, such as testicular torsion, infarction, infection and Fournier gangrene that require prompt surgical intervention.^[31,32]^ Pancreatic hydrocele usually subsides spontaneously once pancreatitis resolves under conservative treatment.^[5,11,19,23]^ However, hydrocele may attribute to congestive varicose vein of testis that increases the risk of male infertility, so treating varicose vein is mandatory for severe cases.^[33,34]^ Although the image demonstrates pancreatic inflammation around right testicular vein, true relationship between pancreatic hydrocele and occlusion of testicular venous flow remains unclear. More cases or studies are needed to clarify the mechanism.

In summary, we presented a case of recurrent pancreatic hydrocele with a novel mechanism. Different from other studies suggesting peripancreatic fluid extending down to the scrotum, our case showed impaired testicular venous flow. It is important to identify this group of pancreatic hydrocele because it is related to infertility. Therefore, pancreatic hydrocele should be carefully examined when evaluating local complications of acute pancreatitis.

## Author contributions

**Investigation:** Yin-Hsi Chang, Ming-Shun Wu.

**Writing – original draft:** Yin-Hsi Chang, Ming-Shun Wu.

**Writing – review & editing:** Yin-Hsi Chang, Shih-Yen Weng, Sheng-Jie Shiue, Chao-Ling Cheng, Ming-Shun Wu.

## Supplementary Material

Supplemental Digital Content
