# Supplementary material for: Hydrocele in recurrent acute pancreatitis caused by testicular venous obstruction: A case report of a rare complication (CARE-compliant)
Source: Medicine (Baltimore). 2020 May 1;99(18):e19738. doi: 10.1097/MD.0000000000019738 (PMC7440237; doi:10.1097/MD.0000000000019738)
Supplement: Supplemental Digital Content [file medi-99-e19738-s001.doc]

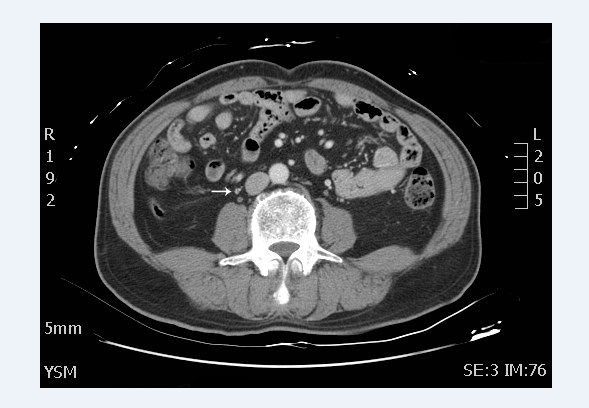


**Supplemental Figure legend**

Abdominal computed tomography scan one month after discharge. An axial view showed that the right testicular vein (arrow) was relief from compression.
